# Vitamin K Deficiency and Thrombophilia in Pregnancy: A Fine Balance Between Bleeding and Thrombus Formation-Insights from a Narrative Review

**DOI:** 10.3390/ijms27135811

**Published:** 2026-06-27

**Authors:** Miruna Samfireag, Ovidiu Potre, Cristina Potre, Ema Borsi, Teodora Hoinoiu, Lavinia Cristina Moleriu, Daniel Pit, Andrei Anghel

**Affiliations:** 1 Department I of Nursing, University Clinic of Clinical Skills, “Victor Babes” University of Medicine and Pharmacy, No. 2 Eftimie Murgu Square, 300041 Timisoara, Romania; samfireag.miruna@umft.ro (M.S.); tstoichitoiu@umft.ro (T.H.); daniel.pit@umft.ro (D.P.); 2 Advanced Cardiology and Hemostaseology Research Center, “Victor Babes” University of Medicine and Pharmacy, No. 2 Eftimie Murgu Square, 300041 Timisoara, Romania; 3 Department of Internal Medicine, University Clinic of Hematology, “Victor Babes” University of Medicine and Pharmacy, No. 2 Eftimie Murgu Square, 300041 Timisoara, Romania; potre.cristina@umft.ro (C.P.); borsi.ema@umft.ro (E.B.); 4 Department III of Functional Sciences, Discipline of Medical Informatics and Biostatistics, “Victor Babes” University of Medicine and Pharmacy, No. 2 Eftimie Murgu Square, 300041 Timisoara, Romania; moleriu.lavinia@umft.ro; 5 Doctoral School, “Victor Babes” University of Medicine and Pharmacy, No. 2 Eftimie Murgu Square, 300041 Timisoara, Romania; 6 Department of Biochemistry, “Victor Babes” University of Medicine and Pharmacy, No. 2 Eftimie Murgu Square, 300041 Timisoara, Romania; biochim@umft.ro

**Keywords:** vitamin K, thrombophilia, pregnancy, protein C, protein S, hemostasis, hypercoagulability

## Abstract

This current research is a narrative review that seeks to establish the occasions under which thrombophilia can result in complications regarding bleeding and thrombosis during pregnancy. Under such circumstances, the influence of vitamin K deficiency is considered, since vitamin K plays an important role in activating the coagulation system. This occurs directly, via the activation of coagulation factors, as well as indirectly, through the activation of proteins S and C. Both proteins play an important role in the hemostatic mechanism of thrombosis and bleeding. However, the risk associated with the relationship between thrombosis and bleeding changes during pregnancy and is heightened by the natural tendency towards hypercoagulability during pregnancy. This paper presents a narrative review of the literature concerning the links between vitamin K, protein C, and protein S in relation to thrombophilia from the perspectives of both biochemistry and medicine, with a special focus on pregnancy. The study examined factors that could be useful to define the balance between hemorrhagic and thrombotic tendency, comparing conventional methods of studying hemostasis with other possible tests that can help better understand the interplay between hemorrhage and thrombosis. Collectively, disorders within the processes associated with vitamin K-mediated blood clotting may have a considerable effect on the woman’s thrombotic risk, especially for women who suffer from thrombophilia. This study confirms the need for monitoring and personalized treatment options to avoid thrombotic and hemorrhagic complications during pregnancy.

## 1. Introduction

Since vitamin K is required for proper blood clotting function, this vitamin earned its name. There is no doubt that vitamin K is essential for blood coagulation [1]. Hence, vitamin K takes its name from the German word for “coagulation” (Koagulation). In recognition of their remarkable achievements in researching vitamin K, Henrik Carl Peter Dam and Edward Adelbert Doisy received the Nobel Prize in Physiology or Medicine in 1943 (awarded in 1944 due to war conditions). Dam identified vitamin K (K for Koagulation) and its functions in blood clotting, whereas Doisy determined vitamin K’s chemistry and made the synthesis possible, which dramatically changed treatment for hemorrhagic conditions, especially in babies [2]. Moreover, the committee considered the importance of vitamin K [1].

The level of vitamin K is clinically important when it comes to blood coagulation problems like thrombophilia. The presence of a tendency to form inappropriate blood clots, called thrombophilia, may be caused by disturbances in coagulation factor activity or their regulation [3]. Vitamin K dysfunction or metabolic alterations can influence the risk of thrombotic events because vitamin K contributes to the activation of numerous anticoagulant and procoagulant proteins, such as proteins C and S [4].

Without sufficient vitamin K, the γ-carboxylation modification, which is vital for the biological function of these proteins, cannot occur. Thus, the physiological systems responsible for the anticoagulative effect are undermined, making the physiological equilibrium between anticoagulation and pro-coagulation processes favor the latter.

The lack of regulation over the coagulation process due to vitamin K deficiency may predispose individuals to thrombosis. The complexity of vitamin K functions in coagulation and vascular physiology is demonstrated by its involvement in the development of thrombophilia despite vitamin K deficiency being more often associated with hemorrhagic disorders [5].

Vitamin K insufficiency is relatively prevalent in certain populations and medical conditions based on epidemiologic data. According to the literature review, the percentage of vitamin K sufficiency among otherwise healthy individuals ranges from 8% to 31% depending on the testing method used [6].

Neonates are especially susceptible; in the absence of preventive supplementation, up to 12% of at-risk newborns may experience early vitamin K deficiency bleeding (VKDB) (within 24 h after delivery), and 0.25% to 1.7% of children may suffer classic VKDB (between 1 and 7 days). With an estimated prevalence of 4.4 to 72 instances per 100,000 infants, late VKDB (from 1 week to 6 months) is less common but more severe, causing significant morbidity [7,8].

Additionally, in population cohorts, functional markers of inadequate vitamin K are raised among individuals, and the prevalence is higher in populations with chronic illnesses and the elderly.

### 1.1. Aim and Objectives

Although the involvement of vitamin K deficiency in thrombophilia is currently under investigation and debate, the importance of vitamin K in clotting has been clearly demonstrated in numerous biochemical studies [6]. While vitamin K deficiency was previously thought to lead to hemorrhagic conditions, recent evidence suggests that it may be related to prothrombotic conditions as a result of changes in the activity of vitamin K-dependent proteins. In our attempts to clarify whether vitamin K deficiency leads to thrombophilia or vice versa, we have initiated this literature review. This narrative review aims to elucidate the complex interplay between vitamin K deficiency and thrombophilia, focusing on the fine balance between bleeding and thrombus formation. The objective of this review is to elucidate how γ-carboxylation alterations and the vitamin K physiology might interfere with the delicate balance of hemostasis and shed light on what it means to consider the balance between bleeding and thrombosis mechanistically in view of the close interaction of vitamin K metabolism with the anticoagulant proteins.

In addition to reviewing the existing data regarding the effects of vitamin K deficiency on the functioning of protein C and protein S and its molecular and metabolic basis, the main objective of this review is to evaluate the extent to which a pro-thrombotic tendency can be associated with vitamin K deficiency regardless of its cause—either dietary or due to warfarin intake or systemic conditions. Another aspect of interest is whether there is enough evidence associating decreased γ-carboxylation of the proteins with increased likelihood of thrombophilia based on experimental and clinical findings, and if so, whether there are any gaps within the current literature.

### 1.2. Methodology

The present study is a narrative literature review that concentrates on the biochemical and clinical connections between vitamin K metabolism, the functions of proteins C and S, and thrombophilia.

Articles with original research, systematic review, narrative review, and clinical study design written in the English language were selected. Preference was given to those that explained the biochemistry of vitamin K-dependent gamma-carboxylation, the physiology of the anticoagulant system involving proteins C and S, and diseases related to a deficiency of vitamin K acquired or congenital. Initial screening of articles was carried out through title and abstract, followed by thorough analysis of the article’s full text to determine if the article was eligible for inclusion or exclusion from the study. Furthermore, the papers selected were grouped according to the level of evidence according to their methodology. The highest levels of evidence included systematic reviews, meta-analysis, and clinical practice guidelines, which were followed by cohort and case-control studies. On the other hand, lower levels of evidence were represented by narrative review and case reports. This grouping helped to highlight the level of the available evidence concerning the interrelationships between vitamin K, proteins C/S, thrombophilia, and pregnancy outcomes.

In addition, the present review considered research related to inter-individual differences in vitamin K-dependent coagulation systems and how they might be significant for personalizing treatment. There was special emphasis on investigations of inter-individual variations in protein C and protein S concentrations, changes in these proteins when they are depleted due to vitamin K antagonists, and formation of the prothrombotic state associated with the use of this type of medication at the early stages of therapy. This line of research was examined as a way to determine whether variability in the biochemical processes involving vitamin K could influence thrombotic risk.

**Figure 1:**
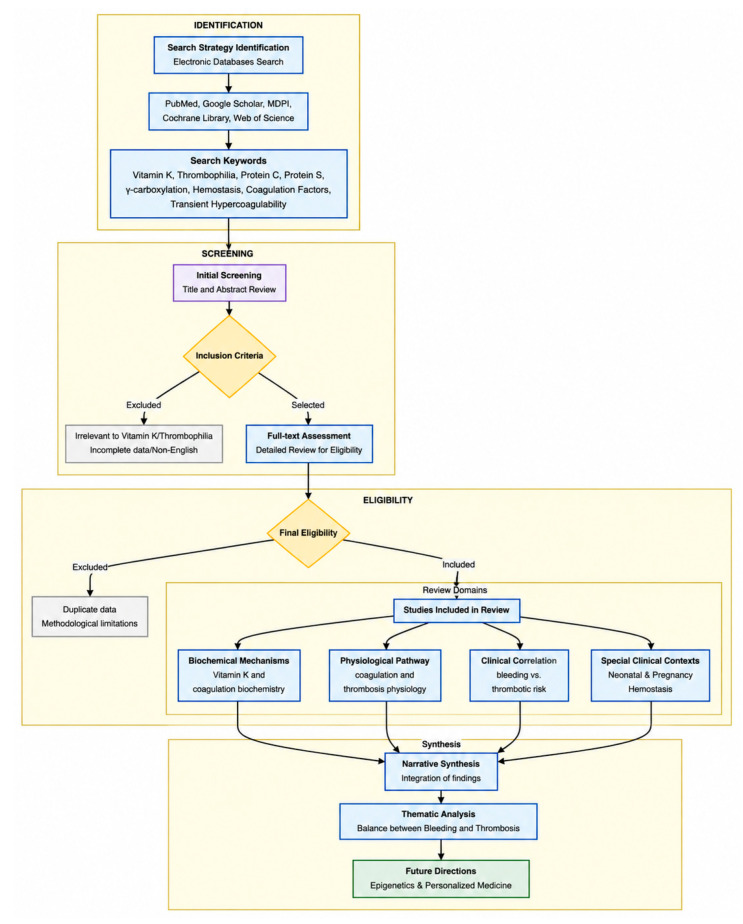
Flowchart of the study design.

The structure of the current narrative literature review relates to the relationship between the metabolism of vitamin K, the role of protein C and S, and thrombophilia and is illustrated in Figure 1 below. The flowchart shows the process followed in the design of the study from the point of selecting literature until the clinical and biochemical synthesis of information.

## 2. Vitamin K, Protein C and Protein S: Biochemistry and Physiology of the Anticoagulant System

For close to half a century, the involvement of vitamin K was believed to be restricted to only one metabolic process, which is hemostasis [9]. Vitamin K and vitamin K-dependent proteins were mostly implicated in the process of blood clotting [10].

Vitamin K is among the fat-soluble vitamins; it consists of a 2-methyl-1,4-naphthoquinone ring that serves as a structural basis for the biological activity of the vitamin [11]. The described ring structure serves as the basic molecular skeleton for all vitamin K derivatives [12]. There are two types of vitamin K: vitamin K1 (phytoquinone or phytonadione) and vitamin K2 (menaquinone) [11].

Moreover, there are several synthetic analogues of vitamin K. The first one is vitamin K3 (menadione), which works as provitamin. Though menadione is widely used for treatment of hemorrhages, it is not recommended for consumption by people since it demonstrates a low level of toxicity and the ability to cause allergic reactions and hemolytic anemia [13]. It turns out that vitamin K4 (menadione ester) has antitumor activity [14].

The indispensable role of vitamin K as a cofactor in the γ-carboxylation of glutamate residues in different proteins is indicated by its characteristic chemical structure with a 2-methyl-1,4-naphthoquinone ring. The physiological role of this important protein modification consists in activating such molecules as clotting factors, anticoagulant proteins, and other proteins which are necessary for bone metabolism and vascular health.

Gla proteins are a set of proteins containing γ-carboxyglutamic acid (Gla) residues. The special amino acid is produced via the γ-carboxylation of specific glutamic acid residues within a protein in a vitamin K–dependent manner [15].

The posttranslational γ-carboxylation of certain proteins through various mechanisms is another essential role played by vitamin K. The implication of vitamin K is its ability to modify specific glutamate residues of such proteins to γ-carboxyglutamate residues. This process, known as γ-carboxylation, is solely dependent on vitamin K as a cofactor. The physiological activity of these synthesized Gla proteins relies solely on the ability of these proteins to complex with calcium ions, which is made possible by the introduction of a new carboxyl group into the glutamic acid residues. The synthesis of Gla proteins, which occurs in the endoplasmic reticulum during the secretion of these proteins on the cell surface or outside the cells, is essential for activating vitamin K–dependent proteins [16].

The important functions of vitamin K-dependent proteins include blood clotting, bone metabolism, and vascular health, indicating the importance of the vitamin in maintaining normal physiological functions [10]. Vitamin K-dependent proteins include coagulation Factors II, VII, IX, and X and anticoagulant factors protein C and protein S [17,18]. Vitamin K-dependent proteins have different plasma half-lives. They include protein C (with the lowest half-life: 6–8 h); Factor VII (4–6 h); protein S (about 30 h); Factor IX (20–24 h); Factor X (24–40 h); and prothrombin (Factor II, 60–72 h). Therefore, in case of vitamin K deficiency and when warfarin is administered, levels of protein C will be decreased faster than those of procoagulant factors. The kinetics of vitamin K-dependent proteins and their role in hemostasis are structured in Table 1 below.

This means that in the situation of vitamin K deficiency or warfarin treatment, protein C drops faster than the coagulation factors do. Thus, the imbalance that occurs may lead to a short-term period when there is a hypercoagulable state because of the total drop in vitamin K-dependent coagulation factor activity. However, when the level of the procoagulant proteins starts to decline, there is an anticoagulant effect dominating the situation. This is how the paradox of thrombosis during vitamin K antagonism is explained [18].

The synthesis of Gla-protein proteins is impossible without the presence of vitamin K. Indeed, the mineralization of bones in jawed vertebrates is regulated by several vitamin-dependent proteins [13]. Among them, matrix Gla protein and bone Gla protein belong to the same gene family due to their high degree of amino acid similarity [19,20].

Several recent studies have shown the role of vitamin K in the development of neurons [21]. A current review has provided several examples of its role in the physiology of the central nervous system and therefore suggested the possibility that deficiency in the vitamin may lead to cognitive impairment [22].

In addition to its traditionally well-known role in hemostasis, recent studies have shown the participation of vitamin K in the physiological processes of the nervous system, paving the way to future investigations of its physiological role in neurological diseases.

Vitamin K functions as a cofactor in post-translational modifications of hemostatic proteins, especially by the introduction of a γ-carboxyl group to glutamate residues. The essential components of the endogenous anticoagulation system, proteins C and S, are glycoproteins dependent on vitamin K and are mainly synthesized in the liver [23,24]. Understanding the pathway would give us a clue as to how we could approach comprehending the intricate balance between clotting and bleeding, since a failure in the γ-carboxylation reaction could swing the pendulum towards bleeding, or even thrombosis.

Protein C is a vitamin K-dependent serine protease synthesized in an inactive form (zymogen) by the liver. After its modification, protein C becomes capable of activation by the action of the thrombin–thrombomodulin complex in the membranes of endothelial cells. Activated protein C (APC) performs an anticoagulant function by inactivation of Factor Va and VIIIa and preventing thrombin synthesis. The structure of protein C consists of three domains: Gla domain, two EGF-like domains, and a protease domain. Another vitamin K-dependent protein called protein S serves as a cofactor of APC and enhances the affinity of APC for phospholipid and substrate interaction. Protein S exists in two forms—the unbound one and C4b-binding-protein bound one. The ratio of both forms is crucial in relation to the efficacy of anticoagulation function performed by the complex protein C/protein S. Inactivation of vitamin K physiology or changes in biosynthesis of Gla-containing proteins influence the binding with calcium ions and reduce the effectiveness of the protein C/protein S complex [24,25].

Vitamin K is one of the necessary factors in the process of carboxylation of certain coagulation factors, which plays an integral role in the mechanism that makes possible their calcium-binding abilities. The most significant among the vitamin K-dependent factors are protein C and protein S, which are involved in the physiological anticoagulant system [24,26].

## 3. New Trends in the Etiology of Thrombophilia Link with Vitamin K Deficiency

Thrombophilia is a genetic defect predisposed to the risk of thrombosis, which may also occur secondary to acquired thrombophilia [27,28]. Modifications of one or several functions of hemostasis, including factors of coagulation, plasma proteins, blood circulation, vessel wall integrity, cellular components resulting in hypercoagulability, cause thrombosis. Venous and arterial thrombosis occur consequently. During treatment of a patient with thrombosis, it would seem reasonable to identify whether his/her hypercoagulability syndrome is of an inherited or acquired nature. Venous or arterial thrombosis is a multifactorial condition that manifests itself as the ultimate clinical sign of a congenital or acquired problem. Asymptomatic patients with a family history of venous thromboembolism (VTE) often request a test for thrombophilia [3].

**Figure 2:**
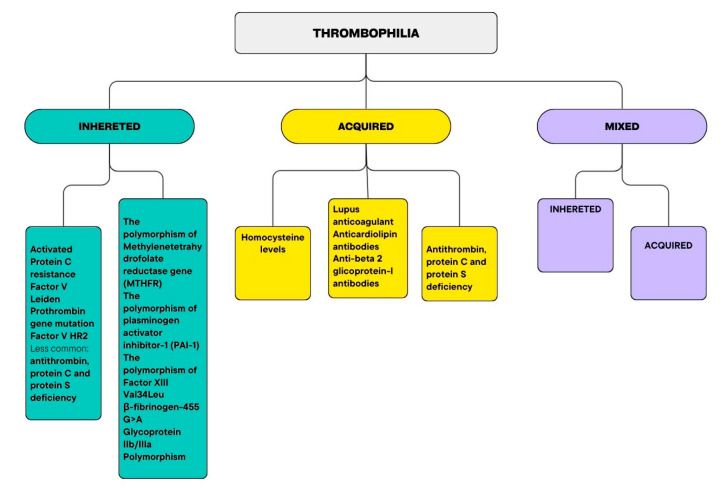
The classification of thrombophilia. Information taken from [29,34,35].

In the 1900s, hereditary thrombophilia was regarded as a rare genetic disease. Later, certain tests for diagnosing hereditary thrombophilia became available. Various risk factors associated with thrombotic complications, including increased age, immobilization, prolonged orthostasis, obesity, nutrition, smoking, elevated estrogen, and contraceptive pill usage (increasing chances of VTE threefold) [29,30]. The classification of thrombophilia is described in Figure 2 below. Thrombophilia refers to a condition that leads to a tendency towards developing blood clots because of hypercoagulability. There are three major types of thrombophilia, namely hereditary thrombophilia, which results from mutations of genes associated with blood clotting; acquired thrombophilia that occurs due to some underlying diseases, including autoimmune disorders, pregnancy, or tumor; and mixed thrombophilia that involves genetic and acquired factors [31,32,33].

Heritable thrombophilia is linked primarily with predisposition to VTE; on the other hand, acquired thrombophilia is related to venous and arterial thrombosis [36]. Inheritance of thrombophilia can be further classified into two types based on increased amounts of coagulation factors including APC resistance, Factor V Leiden mutation, and prothrombin gene mutation in the first type, and reduced amounts of coagulation inhibitors like antithrombin, protein C, and protein S deficiency in the second type.

The polymorphism of Factor V characterized by the presence of HR2 haplotype has been reported to play an independent role in causing APC resistance in both healthy people and those suffering from thrombophilia regardless of the presence of Factor V Leiden mutation. Having the combination of HR2 haplotypes and Factor V Leiden causes greater resistance to APC, thereby creating greater imbalance in the coagulation process. Several research works have revealed that the existence of HR2 haplotypes increases the chances of developing VTE by two to three fold [37].

Polymorphisms of genes associated with the processes of hemostasis and fibrinolysis have been actively studied due to the possibility of their influence on the development of thromboembolic diseases, pregnancy complications, and other cardiovascular pathologies. Among the mutations that have attracted the attention of clinicians, one can single out mutations MTHFR, PAI-1, Factor XIII, β-fibrinogen, and platelet glycoprotein [38,39,40,41,42,43].

Therefore, Factor V Leiden and prothrombin G20210A make up to 70% of all genetically identified forms of thrombophilia. The most severe but rare causes are defects of antithrombin, and protein C and protein S deficiencies [27,34,44].

Genetic variations in MTHFR genes, for example, C677T and A1298C, have been found to affect the metabolism of folic acid and homocysteine. Deficiency in enzyme activity from these genetic mutations may lead to hyperhomocysteinemia, which causes dysfunction in endothelial activity and prothrombotic activity. Genetic variations in MTHFR genes are controversial in their clinical relevance because most studies have proven that these factors have little effect on thrombotic disease incidence [38,45].

PAI-1 gene polymorphism, especially 4G/5G, located in the promoter region, is responsible for PAI-1 protein production, which is a strong inhibitor of fibrinolysis. PAI-1 4G polymorphism has been associated with higher levels of PAI-1 protein, which leads to poor fibrinolysis and increased chances of thrombosis formation. This polymorphism has also been explored in recurrent pregnancy loss and metabolic syndrome [46].

Factor XIII Val34Leu polymorphism has a great effect on fibrin stabilization. First, one should pay attention to the Leu34 variant of this polymorphism, which has been shown to have an increased rate of Factor XIII activation and altered fibrin structure. As a result, this factor makes fibrin clots resistant to degradation. At the same time, several pieces of research have proven that the described polymorphism is considered to have a protecting effect on thrombosis development since it promotes the development of fibrin clots susceptible to fibrinolysis [47].

β-fibrinogen promoter -455 G>A mutation is located in the fibrinogen promoter region, which regulates the level of fibrinogen protein content in plasma. One mutation variant has been associated with increased levels of fibrinogen proteins in plasma and consequently the increased viscosity of the blood makes it risky for thrombosis development. Other factors contributing to the development of the diseases in question are smoking and inflammation [48,49].

Platelet glycoprotein IIb/IIIa is a genetic polymorphism responsible for changes in the fibrinogen receptor located on platelets. The PLA2 (Pro33) allele is associated with increased platelet aggregation and thrombogenesis risk. However, the role of the mentioned allele in arterial thrombosis and myocardial infarction is under debate [50].

While individually each of these polymorphisms has a negligible effect on a person, together, along with various other risk factors in the environment and acquired during life, they may exert a significant effect on the predisposition to thrombosis [51].

Acquired disorders of hemostasis, in addition to hereditary thrombophilia, can lead to hypercoagulability disorders. Acquired disorders that can maintain hypercoagulation due to increased levels of pro-coagulants, decreased levels of anti-coagulants and other disorders of hemostasis include hyperhomocysteinemia, antiphospholipid antibody syndrome, increased level of procoagulants, and decreased anticoagulants [27].

Antiphospholipid syndrome (APS) is an acquired type of thrombophilia condition that is indicated by thrombosis in veins or arteries and is associated with antiphospholipid antibodies (aPL) [52]. APS has two criteria for its diagnosis, including a laboratory criterion that involves the presence of high concentrations of IgM/IgG anti-cardiolipin antibodies, of anti-beta 2 glycoprotein-I antibodies or of lupus anticoagulant, and a clinical criterion of pregnancy or morbidity or severe venous thrombosis. Obstetric problems are also involved [3,53].

Coagulopathies are frequently caused by vitamin K deficiency in people of all ages. Reduced vitamin K is an essential cofactor for the post-translational γ-carboxylation reaction of several vitamin K-dependent proteins, called Gla proteins, that include proteins C and S, and Factors II, VII, IX, and X [54]. Their actions depend on the high-affinity calcium-binding sites that are produced by this carboxylation. Vitamin K1 from food, especially green leafy vegetables, provides half of the daily requirements for vitamin K, a fat-soluble vitamin, while vitamin K2 from gut flora provides the other half. Vitamin K deficiency can develop in a matter of days. Neonates and newborns are most at risk of bleeding due to vitamin K deficiency, unless they get regular vitamin K prophylaxis at birth [55].

A crucial part of the vitamin K-dependent coagulation cascade is protein C. It works as an anticoagulant by deactivating Factors V and VIII. A prothrombotic condition caused by an acquired or hereditary protein C deficit can manifest as anything from asymptomatic to venous thromboembolism [3,56]. Protein S serves as an important cofactor for protein C when it is activated, thus boosting the effect of its anticoagulant properties and being responsible for the modulation of thrombin synthesis. However, apart from its function in the coagulation cascade, this protein demonstrates anti-inflammatory properties and is cytoprotective as well. The lack of protein S either inherited or acquired is a risk factor for thrombosis and thrombotic disorders. Protein C and protein S regulate the delicate balance between procoagulant and anticoagulant mechanisms in physiological conditions [24].

A protein C deficit predisposes a person to thromboembolism by modifying the balance between procoagulant and anticoagulant proteins. The function of naturally occurring anticoagulants is essential to counteract the effects of extended exposure of procoagulant proteins and platelet phospholipids to the vessel wall because of the decreased blood flow velocity in the venous circulation. It could account for the higher risk of pulmonary embolism at a relatively young age and venous thromboembolism, which includes deep vein thrombosis. The precise pathophysiology in this regard is still debatable, even though protein C deficiency seems to be linked to arterial thromboembolism as well [57].

In the conventional sense, vitamin K deficiency and thrombophilia can be thought of as two ends of the hemostatic process—hemorrhage on one hand and thrombosis on the other. Nonetheless, the link between the two is not so straightforward as is often considered [5].

The vitamin K-dependent carboxylation process plays an important role in the formation of γ-glutamic acid residues in proteins that act in the hemostatic system: the procoagulation Factors II, VII, IX, and X and the anticoagulant proteins C and S. Vitamin K deficiency causes a general decrease in the concentrations of all vitamin K-dependent proteins; however, due to the short half-life of protein C, its concentration decreases faster than the concentration of procoagulant factors. Thus, during vitamin K deficiency, the body experiences a temporary hypercoagulable condition [58].

For patients with inherited thrombophilic mutations, such as Factor V Leiden and prothrombin G20210A mutations, vitamin K deficiency may exacerbate the condition of hemostatic imbalance that already exists in these patients [59]. This is because anticoagulant activity decreases as protein C and protein S cannot perform their functions effectively, making this condition a potential factor in thrombotic problems despite the low probability of clot formation due to a lack of coagulation factors [23].

Moreover, vitamin K deficiency may be common in patients with certain conditions (malnutrition, chronic liver disease, prolonged antibiotic therapy). Vitamin K deficiency in intensive care unit patients may result from malnutrition, impaired intestinal absorption, antibiotic therapy, high turnover rate, and genetic predisposition [60]. Hence, thrombosis could develop in a patient because of anticoagulant insufficiency rather than hemorrhage.

Moreover, according to various sources, women with inherited deficiencies of antithrombin, protein C, or protein S are at a higher risk of venous thromboembolism complications during pregnancy and postpartum periods [61,62].

Therefore, deficiency in vitamin K does not automatically result in hemorrhage; rather, it can induce the development of blood clots [26].

## 4. Clinical Implications

Analysis of the hemostatic balance in terms of vitamin K metabolism calls for an approach that looks at the issue from different angles—clinical, biochemical and physiological ones. Vitamin K [4] plays a central role in hemostatic balance because metabolism of this vitamin activates various factors related to the coagulation and anticoagulation processes that are dependent on the vitamin. Proteins C and S are vitamin K-dependent anticoagulant proteins responsible for the prevention of excess formation of thrombin. Any disruption in vitamin K metabolism decreases their effectiveness leading to bleeding tendency or thrombotic complications [24]. Inability to activate these proteins due to disruptions in vitamin K metabolism may bring about an imbalance between procoagulation and anticoagulation mechanisms, causing either excessive bleeding or clot formation. In the clinical setting, investigation into the vitamin K-dependent hemostatic balance begins with assessing the risk factors for vitamin K deficiency. These can include poor nutrition, malabsorption problems, hepatobiliary disease, antibiotics, or use of vitamin K antagonists such as warfarin or acenocoumarin [63]. Warfarin acts as an anticoagulant drug by inhibiting the recycling process of vitamin K, which in turn limits the production of its active form, necessary for carboxylation of proteins. Due to this inhibition, there will be impairment in the formation of active Factor II, VII, IX, X, and anti-clotting factors such as proteins C and S [64,65].

Clinical trials conducted earlier pointed out that there may be a link between poor vitamin K nutrition and a tendency towards being prothrombotic, especially among at-risk individuals. Critically ill patients, including those admitted to intensive care units and those with infections like COVID-19, were found to have poor vitamin K nutrition levels in many instances, leading to abnormalities in their blood clotting profiles and thrombotic manifestations. Such observations have been made in light of the disturbance in the balance of vitamin K procoagulants and anticoagulants, along with disturbances in the activation of extrahepatic vitamin K proteins [9,66,67].

Standard blood testing usually consists of coagulation laboratory analyses, such as prothrombin time, which are more responsive to changes in the amount of vitamin K-dependent coagulation factors. However, coagulation tests alone are not sufficient to assess the hemostasis system. One method for a more advanced assessment of patients with such a disorder includes testing the level and activity of protein C and protein S, which help understand the functioning of endogenous pathways of anticoagulation. If there is any decrease in their activity [24,68], it will be an indication of prothrombotic tendency despite the existence of increased blood clotting times. In some patients, a variety of other functional and whole blood tests (e.g., thrombin generation test) can add valuable information to the understanding of the balance of bleeding and thrombus formation.

Furthermore, the analysis of hemostatic balance during pregnancy may require taking into account a combination of clinical, laboratory, and instrumental markers that can evaluate both thrombotic and hemorrhagic predisposition. Specifically, the use of biochemical markers including serum level of D-dimer and fibrinogen, in conjunction with obstetric and gynecological ultrasound, may provide additional data about endothelial damage, placental vessel changes, and presence of hypercoagulation. Such examinations will help to assess the risk of hemorrhage and thrombosis on an individual basis, especially in women who have thrombophilia either by nature or development [31,69].

Pregnancy is characterized by a unique and dynamic physiological state of hypercoagulability, a vital evolutionary adaptation designed to minimize the risk of life-threatening hemorrhage during delivery and the postpartum period [3,70]. This state is driven by significant shifts in the hemostatic balance, involving a marked increase in the levels of procoagulant factors—specifically Factors VII, VIII, IX, X, and XII—as well as a two- to threefold rise in fibrinogen concentrations, which peak at the time of delivery [3,51,70]. Concurrently, there is a notable reduction in the activity of natural anticoagulants; for instance, free protein S levels significantly decrease, while resistance to activated protein C (APC) often emerges during the third trimester [70,71]. The integrity of the utero–placental circulation is paramount for fetal survival, requiring a delicate equilibrium between pro-thrombotic and anti-thrombotic forces [3,70]. However, the placenta itself constitutively expresses tissue factor, further predisposing the maternal–fetal interface to thrombotic events if the systemic balance is disrupted [71]. A critical factor in this balance is the status of Vitamin K-dependent proteins (VKDPs), which include both procoagulants (Factors II, VII, IX, X) and anticoagulants (protein C, protein S) [18,71]. An important issue in the fetal coagulation system is the poor transport of vitamin K across the placenta, leading to decreased vitamin K content in both fetuses and neonates. As a result, vitamin K proteins are not as abundant in neonates as in other cases. This makes them vulnerable to vitamin K deficiency bleeding disease and increases the effects of vitamin K deficiency or thrombophilia in the mother on the fetal hemostatic process. Moreover, certain aspects of maternal health related to coagulation and anticoagulation treatment during pregnancy could affect fetal development and the duration of pregnancy [72].

In general, a comprehensive strategy for evaluation requires the combination of clinical considerations and specific laboratory tests because they are necessary for reflecting the delicate balance between hemorrhage and thrombosis [73].

Vitamin K deficiency and thrombophilia represent the opposite problems of imbalance in the hemostatic system, both having significant clinical relevance. With decreased activation of clotting factors in vitamin K deficiency, there will be poor clot formation, leading to an increased chance of bleeding that may manifest through mucosal bleeding or even severe cases such as baby vitamin K deficiency bleeding. It is particularly significant for patients with poor nutritional intake, liver problems, and malabsorption because prompt diagnosis and supplementation are vital to avoid life-threatening bleeding situations. Conversely, thrombophilia represents a tendency to form clots, increasing the patient’s likelihood of developing conditions like venous thromboembolism, comprising pulmonary embolism and deep vein thrombosis [26,55].

The importance of screening for those at risk is also emphasized by congenital diseases like Factor V Leiden [74], especially in cases when there is an increased tendency towards the formation of clots, such as during pregnancy or prolonged immobilization. From a clinical perspective, these diseases emphasize the necessity of maintaining the delicate balance in the coagulation process since both too much and too little clotting may be detrimental to health and require accurate assessment and treatment. The connection between vitamin K deficiency and infants is of great significance in clinical practice owing to the high prevalence of vitamin K deficiency bleeding in neonates [75].

As a result of the low concentration of vitamin K in the liver, limited placental transport, and the lack of bacteria that synthesize vitamin K in the gut, babies are vulnerable. This makes them vulnerable to unprovoked bleeding from superficial bruises to life-threatening cases such as brain hemorrhages, as their blood coagulants (II, VII, IX, and X) may be inactive. Since breast milk contains less vitamin K compared to infant formula, this becomes more apparent in babies who solely receive breast milk [7].

In practice, this has seen the common administration of prophylactic vitamin K on delivery, which has considerably reduced the incidence of this life-threatening condition. Neonatal thrombophilia, on the other hand, is less common but nevertheless important, particularly in situations of genetically inherited conditions such as Factor V Leiden, which could pose a risk for neonatal thrombosis under certain situations, such as when using a central venous catheter or when having a serious condition. All the above-mentioned conditions raise questions about the complex neonatal hemostasis system in which bleedings and thrombosis, despite being different, can create significant consequences clinically if neglected [8,76,77].

Regarding the clinical perspective, coagulation disorders are largely affected by factors related to the mother and neonate. The results of pregnancy are largely affected by maternal thrombophilia [70], increasing the chances of developing thrombotic disorders in the neonate and causing placental thrombosis, growth restriction, and adverse perinatal outcomes. Neonatal thrombophilia may be enhanced with inherited genetic factors, such as Factor V Leiden, prothrombin G20210A mutation, and lack of natural anticoagulant factors, including protein C deficiency and protein S deficiency [78,79]. This is especially true when there are additional triggers like prematurity or invasive procedures [70,80].

Compared to all other children, infants are the most prone to thrombosis; critically ill and preterm babies are the most vulnerable groups [81]. The biologically immature hemostatic system of infants, characterized by reduced levels of procoagulants and anticoagulants, further modifies their susceptibility to thrombosis and hemorrhage [78]. Additionally, neonatal thromboembolism, a rare yet deadly condition in pediatric populations, is caused by a combination of at least two prothrombotic triggers in neonates, including central venous lines, infection, and preterm birth [78]. Simultaneously, low maternal vitamin K levels might increase the risk of vitamin K deficiency bleeding, underscoring the significance of prevention measures at birth [82]. To lower morbidity and mortality in this susceptible group, rigorous risk assessment, early diagnosis, and focused intervention are essential due to the interaction between maternal genetic background and neonatal physiological immaturity [79]. While the biochemical role of Vitamin K in the gamma-carboxylation of coagulation factors is well-established, the clinical evidence directly linking vitamin K deficiency to thrombotic events remains complex and, in some contexts, indirect [66,67]. Current observational and experimental research has tried to clarify the problem, making use of parameters like uncarboxylated matrix Gla protein (ucMGP) or des-gamma-carboxyprothrombin (PIVKA-II) [9,67]. There is some evidence that insufficient vitamin K levels relate to increased incidence of cardiovascular problems as well as thrombotic diatheses, especially among people at risk like those with chronic kidney disease or severe infections [67,83,84]. However, it is important to note that many of these associations do not yet prove a direct causal link between isolated Vitamin K deficiency and acute thrombosis, highlighting the need for further large-scale prospective trials [85]. The summary of key clinical studies on vitamin k status and thrombotic/cardiovascular outcomes is structured in Table 2.

## 5. Future Directions

Natural genome modulation is a non-invasive, sustainable, and customized substitute for gene editing in the treatment of chronic diseases, and nutritional epigenetics is a quickly growing field of study. The traditional understanding of vitamin K focuses on its role as a cofactor for the gamma-carboxylation of vitamin K-dependent proteins. However, emerging evidence suggests that vitamin K, particularly the K2 isoform (menaquinone), also functions as a transcriptional regulator, influencing gene expression through the activation of nuclear receptors such as the Steroid and Xenobiotic Receptor (SXR) and its ortholog, the Pregnane X Receptor (PXR) [86,87]. These receptors act as ligand-activated transcription factors that regulate the expression of various genes involved in drug metabolism, transport, and homeostasis. Specifically, vitamin K2 has been shown to bind to and activate SXR/PXR, leading to the upregulation of target genes like CYP3A4, which is involved in the metabolism of several anticoagulants, thereby indirectly influencing the hemostatic balance [87,88].

Furthermore, epigenetic mechanisms, including DNA methylation and histone modifications, play a crucial role in the regulation of hemostatic factors. For instance, the expression of protein S and protein C is subject to epigenetic control, and aberrant DNA methylation patterns in these gene promoters have been associated with an increased risk of venous thromboembolism (VTE) [89,90].

In the context of pregnancy, epigenetic biomarkers are increasingly recognized for their diagnostic potential in thrombophilia-related complications, such as preeclampsia and recurrent pregnancy loss [91].

The great majority of diseases are caused by genetic variations and epigenetic rigidity, which result in aberrant gene expression and differential or aberrant methylation patterns [92]. Dietary decisions made by the general public also affect the environment, which in turn affects epigenetics. For instance, the development of sugar cane contributes to increased methyl group donation and a rise in the frequency of reduced function MTHFR alleles, accounting for 11% of carbon and methane greenhouse gas emissions [92].

The amounts of epigenetic modifiers in immune cells vary [93]. Similar to this, regularity and synchrony in the menstrual cycle and circadian rhythms depend on timely and coordinated fluctuations of epigenetic changes. When applied to the process of hemostasis, recent findings indicate that epigenetics could affect the expression of the vitamin K physiology and the production of vitamin K-dependent clotting factors such as protein C and protein S [94,95]. Dynamic control could impact the differences between people in the efficiency of carboxylation and anticoagulant activity, affecting the delicate equilibrium between procoagulation and anticoagulation pathways. While direct evidence linking vitamin K-mediated epigenetic changes to thrombophilia remains an area of active investigation, the interplay between vitamin K’s transcriptional activity and the epigenetic landscape of coagulation factors provides a plausible mechanism for how nutritional status can influence thrombotic risk [86,96]. Future research focusing on the “nutrigenomic signature” of vitamin K in pregnant women with thrombophilia could lead to more personalized therapeutic strategies.

## 6. Conclusions

The link between vitamin K deficiency and thrombophilia might be found through an integrated research method which would consist of studying not only the issue within the context of the general population but also in terms of pregnancy since the pregnancy state physiologically prolongs the hemostasis into prothrombotic. It seems necessary to conduct population-based studies to find out how vitamin K deficiency relates to higher risks of forming blood clots independently from other factors like malnutrition, chronic infection, hepatic diseases, etc., or together with them.

Additionally, it would be useful to investigate such a relationship in pregnant females to establish whether there are any higher risks for this specific group of people due to their hypercoagulable state during pregnancy.

Secondly, it would be interesting to study further the relationships between vitamin K-dependent prothrombotic and antithrombotic proteins in different physiological states. For example, in an ordinary individual, vitamin K deficiency may lead to relative equilibrium in the level of these two systems; however, in case of pregnancy, where there is physiological downregulation of protein S and upregulation of activated protein C resistance, there may appear imbalance in favor of prothrombotics among patients with predispositions to thrombosis.

Thirdly, research on the impact of vitamin K deficiency in both scenarios, that is, among the general population and pregnant women, is needed. Regarding the general population, research will focus on the effect of endothelial dysfunction and thrombosis. For pregnant women, it should study the function of placental blood supply, especially the risks of micro-thrombosis and inadequate placental perfusion leading to conditions like preeclampsia and fetal growth restriction.

Finally, diagnostic strategies must be improved both in the general population and in pregnant women.

Further studies should therefore investigate the interactions between genetic, epigenetic, and environmental factors that regulate vitamin K homeostasis and the activity of vitamin K-dependent procoagulants, especially proteins C and S [92]. Genetic variants in various elements of vitamin K physiology have been found to affect the individual differences in γ-carboxylation efficacy and anticoagulation efficiency.

Additional research must try to validate markers for vitamin K deficiency and link them to adverse outcomes such as the occurrence of thromboembolism in the general population and pregnancy-related problems in addition to thrombosis. Exploring the connection between vitamin K deficiency and thrombophilia continues to be a dynamic area of investigation that requires not only elucidation of mechanisms but also corroboration in clinical practice.

## Figures and Tables

**Table 1:** The kinetics of vitamin K-dependent proteins and their role in hemostasis. Information taken from [17,18].

Protein	Function	Approximate Half-Life
FACTOR VII (F VII)	Procoagulant	4–6 h
PROTEIN C (PC)	Anticoagulant	6–8 h
FACTOR IX (F IX)	Procoagulant	18–30 h
PROTEIN S (PS)	Anticoagulant cofactor	30–42 h
FACTOR X (F X)	Procoagulant	24–48 h
PROTHROMBIN (F II)	Procoagulant	60–72 h

**Table 2:** Summary of key clinical studies on vitamin k status and thrombotic/cardiovascular outcomes. Information taken from [9,66,67,83,84,85].

Study	Population	Marker/Method	Key Findings	Evidence Level
Dofferhoff et al. (2021) [67]	COVID-19 patients (*n* = 135)	dp-ucMGP levels	Reduced vitamin K status was significantly associated with more severe disease and increased thrombotic complications.	Observational
Shea et al. (2017) [66]	Framingham Heart Study (*n* = 3000+)	Plasma phylloquinone	Lower vitamin K status was associated with higher vascular calcification, a precursor to cardiovascular events.	Prospective cohort
Vermeer et al. (2018) [9]	General population/reviews	Systematic review	Sub-clinical vitamin K deficiency is linked to vascular calcification and potentially increased cardiovascular mortality.	Review/meta-analysis
Ren R. et al. (2021) [83]	Hemodialysis patients (*n* = 50)	Vitamin K2 (MK-7) suppl.	Supplementation improved carboxylation status but did not significantly alter acute coagulation parameters in this small cohort.	Clinical trial (small)
Caluwé et al. (2016) [84]	CKD patients	Vitamin K status	Vitamin K deficiency is identified as a modifiable risk factor for vascular calcification and cardiovascular risk in CKD.	Clinical review/ongoing trial
Theuwissen et al. (2012) [85]	Healthy volunteers	MK-7 supplementation	Low-dose vitamin K2 supplementation influenced the carboxylation of extra-hepatic proteins without inducing hypercoagulability.	Randomized controlled trial

## Data Availability

No new data were created or analyzed in this study. Data sharing is not applicable to this article.

## Abbreviations

The following abbreviations are used in this manuscript:

APC	Activated Protein C
apL	Antiphospholipid antibodies
CKD	Chronic kidney disease
MTHFR	Methylenetetrahydrofolate reductase
PAI-1	Plasminogen Activator Inhibitor-1
PIVKA-II	des-gamma-carboxyprothrombin
PXR	Pregnane X Receptor
SXR	Steroid and Xenobiotic Receptor
ucMGP	Uncarboxylated matrix Gla protein
VKDB	Vitamin K deficiency bleeding
VTE	Venous thromboembolism
